# Paragangliome non sécrétant rétropéritonéal: à propos d'une observation

**DOI:** 10.11604/pamj.2017.26.119.8572

**Published:** 2017-03-02

**Authors:** Yddoussalah Othmane, Jakhlal Nabil, Karmouni Tarik, Elkhader Khalid, Koutani Abdellatif, Ibn Attya Andaloussi Ahmed

**Affiliations:** 1 Service d’urologie B, CHU Ibn Sina, Faculté de Médecine et de Pharmacie de Rabat- Université Mohamed V- Maroc

**Keywords:** Tumeur rétropéritonéale, paragangliome, chirurgie, Retroperitoneal tumor, paraganglioma, surgery

## Abstract

Les paragangliomes rétropéritonéaux non fonctionnels sont des tumeurs rares. Ils sont souvent asymptomatiques et peuvent atteindre des dimensions importantes. Les auteurs rapportent le cas d'une tumeur rétro péritonéale, découverte au cours d'un examen tomodensitométrique réalisé chez une femme âgée de 49 ans en raison de douleurs abdominales non spécifiques. Les formes malignes, plus fréquentes que les formes bénignes, présentent un envahissement locorégional et métastasent tardivement. Le traitement de ces tumeurs nécessite une exérèse chirurgicale la plus complète possible puisque le pronostic en dépend. Il n'existe par contre pas de consensus sur l'utilité des thérapeutiques complémentaires qui peuvent néanmoins constituer un appoint à titre symptomatique.

## Abstract (en)

Non-functional retroperitoneal paragangliomas are rare tumors. They are often asymptomatic and can reach very large sizes. We report the case of a 49-year old woman with retroperitoneal tumor detected during CT scan examination performed to find the cause of non-specific abdominal pain. Malignant forms, more frequent than benign forms, show locoregional invasion and are characterized by delayed-onset metastases. The treatment of these tumors is based on the most complete degree of surgical resection, since prognosis depends on it. There is no consensus on the usefulness of complementary therapies which may nevertheless constitute a supportive treatment.

## Introduction

Le paragangliome ou phéochromocytome extrasurrénalien est une tumeur neuro-endocrine d'origine ectodermique se développant aux dépens des tissus chromaffines. Le siège surrénalien est habituel (90%), la localisation extrasurrénalienne est rare représentant 10% des paragangliomesavec un taux d'incidence de 2-8 cas par million de personnes / année [1]. Ils ont une topographie variable et la forme rétropéritonéale fonctionnelle représente 2% des cas [2]. La forme rétropéritonéale non fonctionnelle est encore plus rare.Leur diagnosticclinique est difficile car elles évoluent lentement. Le potentiel malin de ces tumeurs nécessite le recours àune chirurgie d'exérèse.Nous en rapportons un cas, diagnostiqué par l'étude histologique de la pièce opératoire.

## Patient et observation

**Figure 1:**
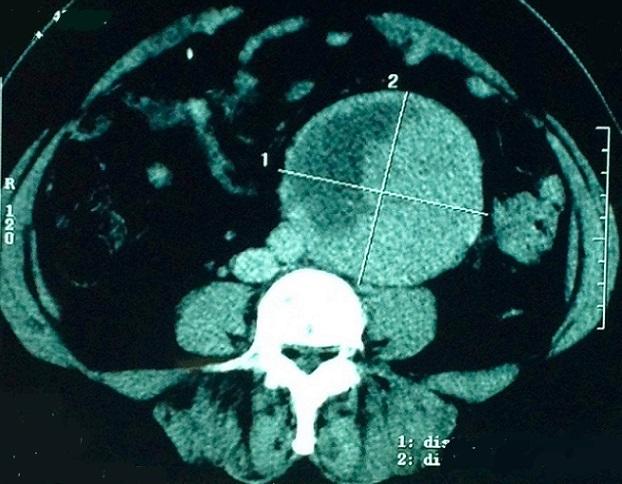
Coupe tomodensitométrique mettant en évidence la masse rétro-péritonéale pseudo encapsule au dessous du pédicule rénale gauche, mesurant 96x88mm et refoulant la VCI et l'aorte vers la droite

**Figure 2:**
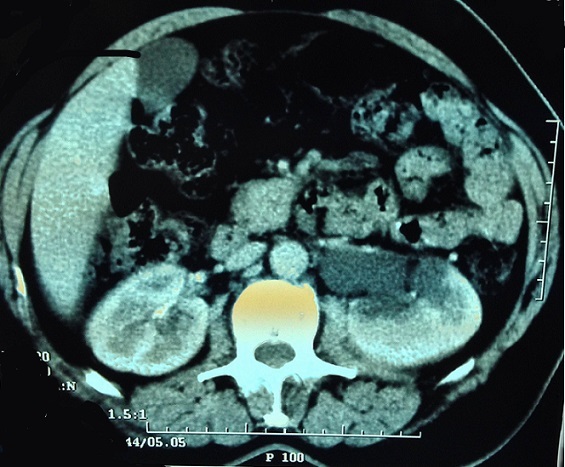
Cliché d'un examen tomodensitométrique montrant l'hydronéphrose gauche secondaire a l'écrasement de l'uretère lombaire homolatérale par la masse RP

**Figure 3:**
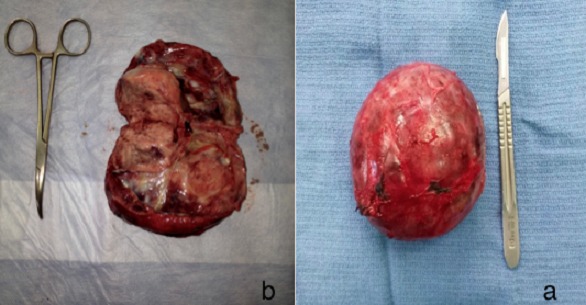
a) Aspect macroscopique: masse tumorale mesurant 120x7x7mm, d aspect kystique et charnue, la paroi est épaissie; b) coupe, aspect jaune acajou avec des zones de nécrose

**Figure 4:**
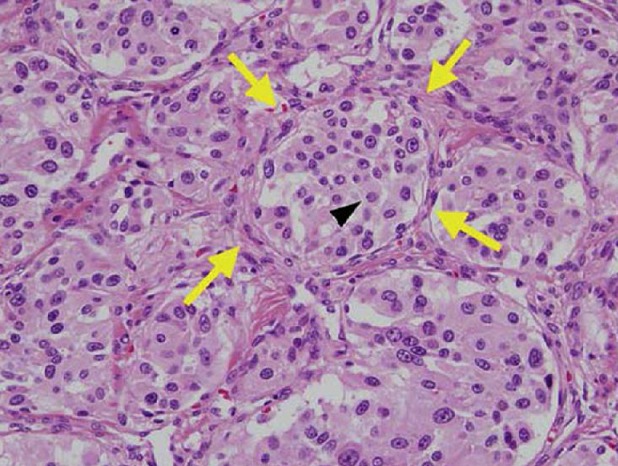
Specthistologique d'une tumeur endocrine centrée sur les vaisseaux avec un très grand polymorphisme cellulaire et demultiples mitoses

E. M., âgée de 49 ans, ayant une hypertension artérielle connue et traitée depuis plus de 10 ans. L'histoire clinique de cette femme débutait par des douleurs abdominales du flanc gauche avec deux épisodes de colique nephretique gauche. L'abdomen était souple, avec une masse palpable du flanc gauche. L'échographie abdominale a révélé l'existence en d' une masse du flanc gauche contenant une composante charnue et une composante liquidien cloisonnée. Avec une hydronéphrose gauche. La tomodensitométrie abdominale, réalisée avant et après injection du produit de contraste, a montré une masse latero aortiquegauche mesurant 96 x 88mm, contenant une composante charnue qui prend le contraste et une composante nécrose (Figure 1), responsable dune compression de l uretère lombaire gauche entrainant une hydronéphrose homolatérale importante (Figure 2). Les dosages biologiques complémentaires avaient éliminé une sécrétion hormonale inhabituelle. Etant donnée la négativité du bilan et le caractère d envahissement loco régional (uretère), une chirurgie d exérèse a étaitdécide par abord para rectale gauche ce qui permettait dedécouvrir une tumeur rétropéritonéale d'environ 90 mmde grand axe, La tumeur était ferme, de contours réguliers, sans capsule. Elle adhérait aux structures du voisinage sans lesenvahir. On réalise alors une exérèse complète de la masse. Les suites opératoires ont été simples. A l'examen macroscopique, la masse pesé 240g mesurant 120x7x7mm, elleétait d aspect kystique et charnue, avec une paroi de kyste qui est épaissie. A la coupe aspect jaune acajou avec des zones de nécrose (Figure 3). L'examen histologique, a montre une proliférationtumorale faite de plage de cordon et d amas se disposant autour de structure vasculaire réalisant une architecture neuroendocrine (Figure 4). Cette analyse morphologique aconclu à un Paragangliome rétropéritonéal. La patiente a été revue en consultation à un mois et sixmois; son examen clinique a été normal et la tomodensitométrie abdomino-pelvienne réalisée après six mois n'apas montré de récidive.

## Discussion

Les tumeurs rétropéritonéales primitives de l'adulte formentun groupe hétérogène de lésions, malignes dans 80 % des cas [3]. Parmi elles, les paragangliomes sont rares. Ils sont développés aux dépens des cellules ectodermiques du système nerveuxautonome, ou de vestiges des tissus chromaffines primitifs setrouvant le long du squelette axial et dans la médullaire de la glande surrénale [4]. Deux pour cent des paragangliomessont rétropéritonéaux. Quatre-vingt-dix pour cent d'entre euxsont d'origine surrénalienne, constituant le groupe des phéochromocytomes. Les 10 % restants proviennent du système nerveuxautonome. Dans ce cas, leur localisation préférentielle est para aortique (43% des cas) [4]. Ils sont généralement vus dans les deuxième et troisième décennies et les deux sexes sont touchés de la mêmemanière [5]. Ces tumeurs sont non sécrétantesdans 40% des cas, ce qui explique à la fois l'absence de signe. Fonctionnel spécifique (pas de sueur, pas de céphalée, de tachycardie, ou d'hypotension) et les difficultés rencontrées dans lediagnostic de cette affection. Notre observation résume parfaitementles caractéristiques principales des paragangliomes. Leurévolution est lente, pauci-symptomatique. Leur découverte estsouvent fortuite ou tardive, la plupart du temps lorsque la masseest déjà palpable. L'association avec une adénomatose endocriniennemultiple (NEM) n'est pas fortuite, certaines formes s'inscrivantdans un contexte familial. Cette éventualité reste cependantrare, tout comme les formes multiples (16,5% des cas) ou associé à une phacomatose [6].

L'imagerie médicale (échographie, tomodensitométrie, IRM) pose le diagnostic de tumeur rétropéritonéale.Les paragangliomes peuvent prendre denombreux aspect en imagerie [7]. Ils peuvent être uniquement tissulaires, contenir de la graisse ou se calcifier partiellement. Certaines tumeurs peuvent se nécroser avec des niveaux liquide-liquide hémorragiques ou donner un aspect de masse kystique avec capsule fibreuse. Les tumeurs de plus de 7cm sont le plus souvent de densité hétérogène. Il n'y a pas de spécificité dans le type de rehaussement. L'injection d'un produit de contraste iodé n'entraîne pas de crise hypertensive et n'est donc pas contre indiquée en cas de suspicion de paragangliome [8]. Les aspects sont également multiples en IRM. En l'absence d'hypersécrétion de catécholamines, il n'y a en préopératoire aucune indication à réaliser une scintigraphie à la méta-iode-benzyle-guanidine (MIBG) [9]. Par contre, cet examen serait positif dans beaucoup de paragangliomes non fonctionnels [9, 10]. En revanche, elle trouve une place prépondérante dans la surveillance post opératoire où elle permet la détection des récidives ou métastases [9, 11]. La sensibilité de la scintigraphie au MIBG est estimée entre 85 et 90% [4], mais elle peut aussi être révélatrice de tumeurs dont l'origine embryologique est proche de celles des paragangliomes (neuroblastes, ganglioneuromes, carcinoïdes, cancers médullaires de la thyroïde) [4, 12]. Malheureusement, il n existe pas de critères fiable clinique, biochimique ou histologiques pour distinguer les formes maligne des formes bégnines. Seulement l invasion loco régional et la présence de métastases à distance du poumon, du foie, et des os ont été utilisés comme des indicateurs de malignité. Selon la littérature seulement 20% des paragangliomes étaient métastatique [1].

La chirurgie représente la base du traitement de ces tumeurs en raison de leur potentiel malin [9]. L'exérèse, qui doit être totale pour être curative, nécessite parfois une extension aux organes adjacents [4, 11, 10]. La possibilité de pratiquer cette chirurgie de manière radicale est estimée à 75% des cas [4, 2]. Dans certains cas, une embolisation préopératoire a pu être proposée, permettant de réduire la vascularisation Tumorale [9, 11]. La célioscopie reste une voie d'abord appropriée pour des lésions de moins de 5 cm [13]. Des thérapeutiques complémentaires peuvent être associées: chimiothérapie, radiothérapie externe ou utilisation d'iode 131 associé au MIBG [4]. La radiothérapie trouve sa place à titre antalgique dans les métastases rachidiennes ou dans le but de stériliser en postopératoire Des reliquats tumoraux [4, 9]. La chimiothérapie peut être envisagée dans les formes métastatiques et comprend en général une association de Décarbazine, Vincristine et Cyclophosphamide [4, 9]. Le Cisplatinium a également donné des résultats encourageants [9]. Ces thérapeutiques adjudantes donnent une réponse positive dans environ 50% des cas, mais n'influencent pas le pronostic de manière significative [4, 10].

## Conclusion

Les paragangliomes Rétropéritonéale extra-surrénalien sont des tumeurs neuroendocrines relativement rares. Le diagnostic de paragangliome non sécrétant est difficile car il n’y a pas de signe clinique et biologique spécifique permettant de faire un diagnostic précoce. L’exérèse chirurgicale constitue le traitement de choix, les thérapeutiques complémentaires constituent surtout un appoint à visée symptomatique.
